# Complete Genome Sequence of the Thermophilic and Facultatively Chemolithoautotrophic Sulfate Reducer *Archaeoglobus sulfaticallidus* Strain PM70-1^T^

**DOI:** 10.1128/genomeA.00406-13

**Published:** 2013-07-05

**Authors:** Runar Stokke, William Peter Hocking, Bjørn Olav Steinsbu, Ida Helene Steen

**Affiliations:** Department of Biology, Centre for Geobiology, University of Bergen, Bergen, Norway

## Abstract

Dissimilatory sulfate-reducing archaea of the genus *Archaeoglobus* display divergent preferences in the use of energy sources and electron acceptors. Here we present the complete genome sequence of the thermophilic *Archaeoglobus sulfaticallidus* strain PM70-1^T^, which distinctly couples chemolithoautotrophic growth on H_2_/CO_2_ to sulfate reduction in addition to heterotrophic growth.

## GENOME ANNOUNCEMENT

The thermophilic euryarchaeon *Archaeoglobus sulfaticallidus* type strain PM70-1 belongs to the family *Archaeoglobaceae* and was isolated from black rust present on the steel surface of a borehole observatory on the eastern flank of Juan de Fuca Ridge, eastern Pacific Ocean (1, 2). The genus *Archaeoglobus* consists of five species with validly published names, *A. fulgidus* (3), *A. profundus* (4), *A. veneficus* (5), *A. infectus* (6), and *A. sulfaticallidus* (1), all of which were isolated from marine hydrothermal systems. A formal species description has not been published for “*A. lithotrophicus*,” isolated from deep oil reservoirs (7).

All members of the genus *Archaeoglobus*, with the exception of *A. profundus* and *A. infectus* (1, 3–6, 8), are capable of chemolithoautrotrophic growth on H_2_/CO_2_ and thiosulfate. However, *A. sulfaticallidus* differs from the other members of the genus *Archaeoglobus* by being able to couple chemolithoautotrophic growth on H_2_/CO_2_ to sulfate reduction (1). This trait has so far been reported only for “*A. lithotrophicus*,” which, in contrast to *A. sulfaticallidus*, is not capable of heterotrophic growth (7).

Whole-genome sequencing was performed using a mixed library of shotgun and 8-kb paired-end reads from a Roche 454-sequencing platform at the Norwegian Sequencing Centre in Oslo, Norway (http://www.sequencing.uio.no). Pyrosequencing reads were assembled using the Newbler assembler version 2.8 (Roche). The Newbler assembly contained 20 contigs in a single scaffold. The genome was finished by filling gaps between contigs with Sanger sequencing of targeted PCR products using an ABI 3730 sequencer (Applied Biosystems). The final assembly comprises 386,142 reads that provide 50× coverage of the genome. Gene predictions and functional assignments were performed within the Integrated Microbial Genomes—Expert Review (IMG-ER) platform (9) in combination with manual curation. Pfam families were categorized according to Pfam27.0, using the command line version of HMMER3 (10, 11).

The complete genome of *A. sulfaticallidus* PM70-1 comprises one circular chromosome with a total size of 2,076,931 bp and a GC content of 43.24%. The genome contains 2,216 protein-coding genes, 1 rRNA operon (one copy each of 5S, 16S, and 23S rRNA genes), and 51 tRNA genes, in addition to 6 other structural RNA genes. Similar to those in the genomes of *A. fulgidus* (12), *A. profundus* (13), *A. veneficus* (Genomes Online Database [GOLD] identification no. Gc01707), and *Ferroglobus placidus* (14), the 5S rRNA gene is not found adjacent to the 16S and 23S rRNA genes. Of the 2,216 predicted coding genes, 1,494 (67.4%) were assigned to known protein functions.

A complete set of genes for the acetyl coenzyme A (acetyl-CoA) pathway of methanogens, which may function as both the core reductive and oxidative carbon metabolic systems, was identified in the genome of *A. sulfaticallidus* (8, 15). Like other members of the *Archaeoglobaceae, A. sulfaticallidus* lacks genes for terminal methanogenesis from acetyl-CoA. Instead, the genome carries a full set of genes for dissimilatory sulfate reduction, common to a wide range of sulfate-reducing organisms (16).

### Nucleotide sequence accession number.

The complete genome sequence of *A. sulfaticallidus* PM70-1^T^ has been deposited in GenBank under accession number CP005290.
